# The Epidemiological Features of the SARS-CoV-2 Omicron Subvariant BA.5 and Its Evasion of the Neutralizing Activity of Vaccination and Prior Infection

**DOI:** 10.3390/vaccines10101699

**Published:** 2022-10-11

**Authors:** Dandan Tian, Wenjian Nie, Yanhong Sun, Qing Ye

**Affiliations:** Department of Clinical Laboratory, The Children’s Hospital, Zhejiang University School of Medicine, National Clinical Research Center for Child Health, National Children’s Regional Medical Center, Hangzhou 310052, China

**Keywords:** COVID-19, SARS-CoV-2, Omicron, sublineages, BA.5, vaccine efficacy

## Abstract

From December 2021 to May 2022, the Omicron BA.1 and BA.2 subvariants successively became the most dominant strains in many countries around the world. Subsequently, Omicron subvariants have emerged, and Omicron has been classified into five main lineages, including BA.1, BA.2, BA.3, BA.4, BA.5, and some sublineages (BA.1.1, BA.2.12.1, BA.2.11, BA.2.75, BA.4.6, BA.5.1, and BA.5.2). The recent emergence of several Omicron subvariants has generated new concerns about further escape from immunity induced by prior infection and vaccination and the creation of new COVID-19 waves globally. In particular, BA.5 (first found in southern Africa, February 2022) displays a higher transmissibility than other Omicron subvariants and is replacing the previously circulating BA.1 and BA.2 in several countries.

## 1. Introduction

On 26 November, the WHO defined B.1.1.529 (first detected in South Africa, 14 November 2021) as the fifth variant of concern (VOC) and named it Omicron [1]. During the past nine months, several identified Omicron subvariants have rapidly spread globally. As of August 2022, Omicron includes five main lineages, including BA.1 [1], BA.2 [2], BA.3 [3], BA.4 [4], and BA.5 [4]. All Omicron lineages have at least 50 mutations accumulated throughout the genome [5] and are the most highly mutated variant containing 31–37 mutations in the spike protein compared to the previous VOCs (Alpha [6], Beta [7], Gamma [8], and Delta [9,10]).

Since April 2022, BA.4 and BA.5 have rapidly replaced BA.2 and have initiated the fifth COVID-19 wave, accounting for more than 50% of sequenced cases in South Africa [4,11]. Several studies have reported that BA.5 exhibits a higher transmission advantage than BA.2 and increased evasion from neutralization antibodies elicited by vaccination and prior infection [4,11,12,13,14,15]. Omicron BA.1 was displaced by BA.2, which in turn was displaced by BA.5, becoming the dominant strain in many regions [12,16,17]. As of 6 June 2022, it is noteworthy that more than 80% of the BA.5 sequences are found in the United States (33.25%), European countries, and South Africa [12]. The current review article aims to analyze the characteristics of key spike mutations, epidemic characteristics, and immune evasion of Omicron BA.5. We hope to provide a scientific reference for monitoring, control measures, and vaccine development strategies for the current or further Omicron subvariants.

## 2. Methods

**Figure 1:**
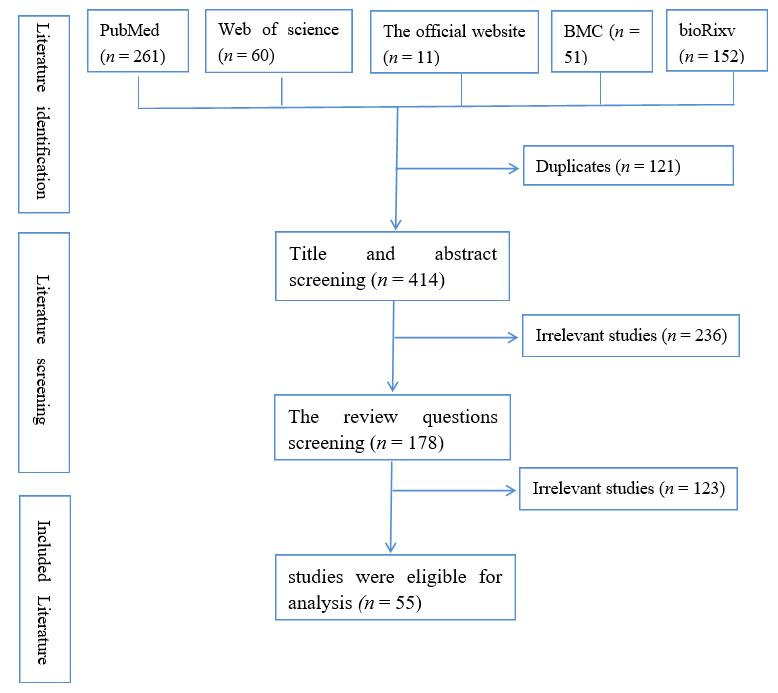
The process of selecting eligible studies flow by the PRISMA diagram depicts.

We used prominent search engines, namely Web of science, PubMed, bioRxiv.org, Google Scholar, BMC and the official website (https://www.who.int/ (accessed on 22 February 2022); https://www.ecdc.europa.eu/en (accessed 17 July 2022); https://www.nicd.ac.za/diseases-a-z-index/disease-index-covid-19/sars-cov-2-genomic-surveillance-update/ (accessed on 28 July 2022); https://covid.cdc.gov/covid-data-tracker/#variant-proportions (accessed on 14 July 2022); and https://weekly.chinacdc.cn/en/article/doi/10.46234/ccdcw2022.104 (accessed on 17 May 2022)). The search keywords were SARS-CoV-2 variant, Omicron, spike mutations, COVID-19 vaccine, immune escape. We removed all duplicates, then screening the literature, which consisted of title, abstract, and full-text screenings, the review question (Does the study focus on SARS-CoV-2? Does the study focus on SARS-CoV-2 variant? Does the study focus on Omicron variants? Does the study focus on Omicron BA.5 spike mutation? Does the study present the Virological characteristics of the Omicron BA.5? Does the study present the infectivity and clinical outcomes of the SARS-CoV-2 Omicron BA.5? Does the study provide the neutralization activity of vaccination and prior infection and some antiviral drugs against BA.5?), yielded 55 final publications for this review, as shown in Figure 1.

## 3. The Characteristics of SARS-CoV-2 Omicron BA.5 Spike Mutations

**Figure 2:**
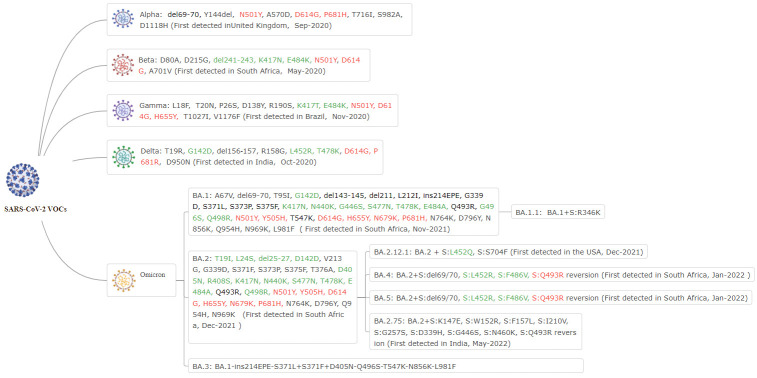
The characteristics of spike mutations of five SARS-CoV-2 variants of concern (VOCs). Notes: Red mutations—these mutations increased the binding affinity and tightness of SARS-CoV-2 spike to ACE2 or increased cell–cell fusion; Green mutations—these mutations reduced neutralization by mAbs, convalescent plasma, and vaccines.

The SARS-CoV-2 Omicron BA.5 (22B/GRA) contains 31 amino acid substitutions and five deletions in the spike protein, including T19I, L24S, del25-27, del69-70, G142D, V213G, G339D, S371F, S373P, S375F, T376A, D405N, R408S, K417N, N440K, G446S, L452R, S477N, T478K, E484A, F486 V, Q498R, N501Y, Y505H, T547K, D614G, H655Y, N679K, P681H, N764K, D796Y, Q954H, and N969K (https://www.nicd.ac.za/diseases-a-z-index/disease-index-covid-19/sars-cov-2-genomic-surveillance-update/ (accessed on 17 May 2022)). It has been reported that T19I, L24S, del25–27, and G142D have caused significant evasion from the N-terminal domain (NTD)-targeted neutralizing antibodies (nAbs) [18]. Additionally, BA.5 has more substitutions mutations (approximately eighteen) located in the receptor-binding domain (RBD) than the previous VOCs [5,10], as shown in Figure 2.

**Figure 3:**
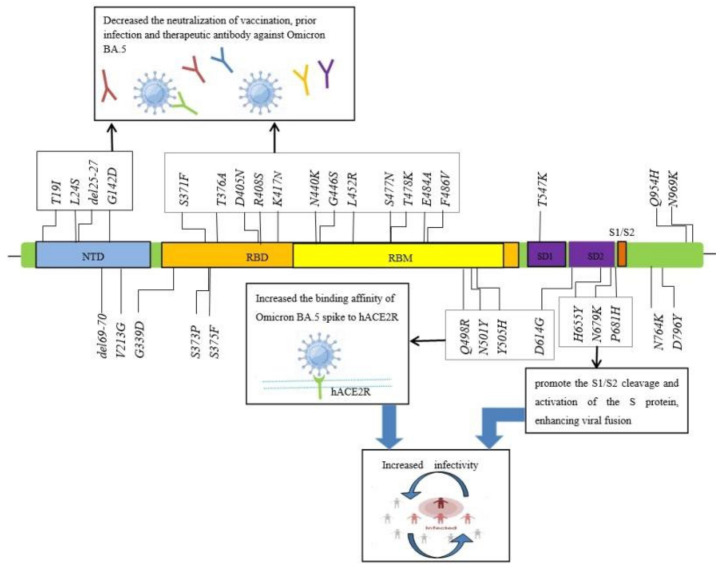
The biological characteristics of the spike mutations of the SARS-CoV-2 Omicron BA.5 variant.

Both pseudovirus experiments and structural modeling indicate that mutations (K417N, N440K [19], G446S, S477N [20], T478K [21], E484A [22], G496S, N501Y [23], Q498R [24], and Y505H, all of which were located in the BA.5 spike) increased the transmissibility of the SARS-CoV-2 variants by increasing the binding affinity and the tightness of RBD to human angiotensin-converting enzyme 2 (hACE2) [25,26] or generated the immune escape of the SARS-CoV-2 variants [27,28,29], as shown in Figure 3. Additionally, BA.5 has also “H655Y + N679K + P681H” mutations located at the S1/S2 border, which could significantly promote the S1/S2 cleavage and activation of the S protein, thus enhancing viral fusogenicity [30,31,32]. Compared with the previous VOCs, BA.5 has some unique mutations (S371F, T376A, D405N, R408S, and G446S) that lie at the edge of the ACE2 interaction sites, and these distant mutations reduce the neutralization of Omicron lineages through certain therapeutic antibodies [33,34,35].

BA.5 shares common mutations in the spike with BA.2, except for del69-70, L452R, F486 V, and R493Q, a reversion mutation [36]. Structural analysis indicated that L452R/Q conferred resistance largely to class 2 and 3 RBD mAbs and affected the sensitivity to vaccine-induced neutralizing antibodies, especially L452R [37]. It has been reported that the L452R/Q substitution renders ~2–5-fold resistance to cilgavimab, which is also antiviral against BA.2 [37].

Notably, F486 V decreased the binding activity of BA.5-RBD to hACE2 due to a reduced hydrophobic interaction, while the R493Q reversion mutation restored a hydrogen bond with H34 and avoided charge repulsion by K31 and increased the affinity between BA.5-RBD and hACE2, thus restoring receptor affinity and consequently the fitness of BA.5 [38]. Additionally, two recent reports claimed that BA.5-RBD showed higher binding affinity to hACE2 than BA.1 and BA.2 due to L452R and R493Q reversion [15]. Additionally, it was reported that F486 V broadly caused steric hindrance to binding by class 2 RBD mAbs, such as REGN10933 and LY-CoV555 [38].

Altogether, Omicron BA.5 has critical spike mutations that were previously reported in other VOCs (Alpha, Beta, Gamma, Delta, BA.1, and BA.2). Compared to BA.2, BA.5 has unique mutations, including L452R, F486 V, and R493Q, which can significantly affect the biological characteristics of BA.5, including increasing transmissibility and causing more immune evasion, as shown in Figure 3.

## 4. The Virological Characteristics of the SARS-CoV-2 Omicron BA.5

Cell–cell fusion experiments showed that BA.5 exhibited a higher propensity for fusion, with an average syncytia area 2.1-fold higher than that of BA.1 and BA.2 [39]. Similar to the fusion results, BA.5 could increase the S processing phenotype owing to the L452R mutation, and BA.5 exhibited comparable surface S expression, which was 1.4-fold higher than that of WT. Furthermore, the analysis of purified viral particles demonstrated that BA.5 increased S1 signals in purified virions compared to BA.2 [39] (with a similar intensity of p24 in the virions).

Pseudovirus infectivity experiments showed that the infectivity of BA.5 was 18.3-fold higher than that of BA.2 and that BA.5 was more efficiently replicated in human alveolar epithelial cells than BA.2, with the levels of viral RNA in the supernatant of rBA.5-infected cultures being 34-fold higher than those in rBA.2-infected cultures [40].

These results suggest that BA.5 exhibited higher fusogenicity and increased spike processing and that BA.5 has a higher transmission advantage than BA.2. In particular, the risk of BA.4 and BA.5 for global health is potentially higher than that of BA.2.

## 5. SARS-CoV-2 Omicron BA.5 Spread Faster in Many Countries and Has a Low Risk of Severe Clinical Outcomes

SARS-CoV-2 Omicron BA.5 was first detected in specimens in February 2022 in South Africa. According to the data from the National Institute for Communicable Diseases (NICD), BA.1 was the predominant strain in January (55%), and BA.2 dominated in February (86%) and March (78%). Subsequently, BA.4 and BA.5 have rapidly replaced BA.2, reaching more than 50% of sequenced cases in South Africa by the first week of April 2022. Sequence analysis showed that the proportion of confirmed cases of BA.4 decreased from 64% (1652/2562) to 29% (79/273), while the BA.5 variant increased from 29% (743/2562) to 67% (182/273) from May to July in South Africa. (Available online: https://www.nicd.ac.za/latest-confirmed-cases-of-covid-19-in-south-africa/ (accessed on 17 May 2022)).

On 12 May 2022, the European Centre for Disease Prevention and Control (ECDC) reclassified BA.5 from variants of interest (VOI) to VOC [41]. According to data from the ECDC, BA.5 accounted for ~37% of the positive cases in Portugal as of 8 May 2022 [41]. As of 14 August 2022, 98.7% (94.4–100.0% from 10 countries) for BA.4/5, 1.1% (0.2–5.6% from nine countries) for BA.2, 0.6% (0.2–0.7%, 44 detections from four countries) for BA.2+L452X, and 0.2% (0.1–0.3%, 35 detections from five countries) for BA.2.75 in the total COVID-19 confirmed sequence in ECDC (Available online: https://www.ecdc.europa.eu/en/publications-data/data-daily-new-cases-covid-19-eueea-country (accessed on 17 May 2022)). Similarly, the number of sequenced episodes of BA.5 increased from 9.5% on 4 June to 66.8% on 2 July in the UK. (Available online: https://www.gov.uk/government/news/covid-19-variants-identified-in-the-uk (accessed on 17 May 2022)). In the USA, BA.5 increased from 9.5% to 56.4% between 4 June and 11 June 2022. The sequence of BA.5 accounted for 88.8% of the total sequence, 5.3% for BA.4, 5.1% for BA.4.6, and 0.8 for BA.2.12.1 as of 14 August 2022 (Available online: https://covid.cdc.gov/covid-data-tracker/#variant-proportions (accessed on 17 May 2022)).

These data highlighted that the number of BA.5 cases is rising worldwide and is becoming the dominant lineage, thus replacing BA.2.

It has been demonstrated that the viral load in the lungs infected with BA.1 is lower than that in the nasal airway [42], and BA.2 is more poorly replicated in CaLu-3 cells than in primary human nasal epithelial cells [43]. One study suggested that the BA.4/5 and BA.2.12.1 variants may retain the reduced pathogenicity of the BA.1 variant [39]. Epidemiologic surveillance [44,45,46,47] revealed that the risk of severe hospitalizations (admission to intensive care or mechanical ventilation or oral/intravenous steroid prescription) and deaths did not increase with the number of COVID-19 cases following the emergence of the Omicron variant globally, with a 20–80% reduction in risk of hospital admission compared to the WT and other VOCs. One clinical study revealed that, after controlling for factors associated with hospitalization and severity (age, sex, presence of comorbidity, previous SARS-CoV-2 infection, and SARS-CoV-2 vaccination status), the adjusted hazard ratio [aHR] of severe hospitalization or death of patients infected with BA.5 was 1.12 (95% confidence interval [CI]: 0.93–1.34) [48], as shown in Table 1. Although infection with BA.5 has a lower risk of severe clinical outcomes, the very higher transmission advantage poses overwhelming challenges to global healthcare systems.

## 6. Omicron BA.5 Exhibits Stronger Neutralization Evasion Than BA.2 against nAbs Elicited by Vaccination and Prior Infection and Some Antiviral Drugs

Pseudovirus neutralization assays claimed that compared to BA.2 and BA.2.12.1, BA.5 showed substantially greater neutralization resistance to two class 2 RBD mAbs (ZCB11 and COV2-2196) as well as modest resistance to two class 3 RBD mAbs (REGN10987 and COV2-2130) [38].

Moreover, the neutralization testing of live viruses showed that the monoclonal antibodies casirivimab and sotrovimab and casirivimab and imdevimab lost neutralizing activity against BA.5 [49]. Compared with BA.1, sotrovimab is less active against BA.5, with a 2.7-fold reduction [36]. BA.5 has been found to be 4–20-fold more resistant to cilgavimab and evusheld than BA.2 [15,50]. The neutralizing activity of the combination of casirivimab and imdevimab against BA.5 was 317.8-fold lower than that against WT [51]. However, cilgavimab, imdevimab, and bebtelovimab neutralized BA.5 [51].

Compared to the WT, BA.5 encoded the P314 L and the P3395H mutation in its RNA-dependent RNA polymerase and its main protease, respectively. In vitro, 50% inhibitory concentration (IC50 with higher values indicating reduced susceptibility) of nirmatrelvir (an inhibitor of the main protease of SARS-CoV-2), molnupiravir, and remdesivir (an inhibitor of the RNA-dependent RNA polymerase of SARS-CoV-2) against BA.5 was 1.6-, 1.5-, and 1.2-fold higher than against WT, respectively [51].

One report published in *Nature* from a team of researchers from Columbia University reported that the nAbs elicited by BA.1 infection after vaccination could neutralize both WT and BA.1 but are largely evaded by BA.5 owing to D405N, L452R, and F486 V mutations [15]. Pseudovirus neutralization assays showed that the 50% neutralization titer (NT50) of plasma from individuals who had received three doses of CoronaVac (2-dose CoronaVac+ZF2001 or 3-dose CoronaVac+BA.1) against BA.5 was reduced by 1.62-, 2.27-, and 4.3-fold compared with BA.2 and was reduced by 1.62-, 2.39-, and 7.98-fold compared with BA.1, respectively [15].

Similarly, the median neutralizing antibody titer (GMT) six months after the initial two doses of BNT162b2 vaccination was 124 against WT but less than 20 against all the Omicron subvariants [16]. Two weeks after the booster, the GMT against BA.5 had decreased by 21.0- and 3.3-fold compared with WT and BA.1, respectively. Moreover, the GMT of plasma from individuals infected with BA.1 or BA.2 after vaccination against BA.5 was 18.7- and 2.9-fold lower than WT and BA.1, respectively [16]. Xie Xuping and colleagues indicated that the GMTs of the four doses of Pfizer or Moderna mRNA vaccine, two doses of vaccine + BA.1-infected sera and three doses of vaccine + BA.1-infected sera against BA.5 were 3.76- (GMTs: 95 vs. 236), 6.22- (GMTs: 274 vs. 1705), and 6.86-fold (GMTs: 297 vs. 2038) lower than those of BA.1, respectively [52].

In addition to the inactivated vaccine and mRNA vaccine, the GMTs of two doses of NVX-CoV2373 (a protein nanoparticle vaccine) were the highest against the WT (GMT: 1401), with 8.1-, 41-, and 30-fold reductions against Beta, BA.1, and BA.4/BA.5, respectively [53]. One month after the third dose of the NVX-CoV2372 vaccine, the GMTs against the WT increased to 10,862, with a significant 10-, 35-, and 12-fold increase in titers against beta (GMT: 1733), BA.1 (GMT: 1197), and BA.4/BA.5 (GMT: 582), respectively, although titers were 6- to 18-fold lower than WT [53].

Currently, several studies have demonstrated that BA.5 could further escape from the immunity induced by vaccination and BA.1 or BA.2 infections, resulting in numerous reinfections in many countries [12,15,39,54,55]. However, prior infection and vaccination were strongly protective against severe hospitalization or death, with (aHR 0.29, 95% CI: 0.24–0.36), (aHR 0.17, 95% CI: 0.07–0.40), (aHR 0.37, 95% CI: 0.33–0.42), and (aHR 0.26, 95% CI: 0.21–0.32) for prior infection, boosted, two doses, and single dose, respectively [48].

## 7. Conclusions

BA.5 is becoming the dominant strain, replacing BA.2 and spreading rapidly in many countries, and will shortly cause the next COVID-19 wave. BA.5 has a much higher transmission advantage than other Omicron subvariants, leading to challenges to global healthcare systems. In the future, more attention should be given to future evolutionary directions and strategies for developing next-generation vaccines and therapeutics.

## Figures and Tables

**Table 1:** The epidemiological characteristics of the SARS-CoV-2 Omicron BA.5 variant.

WHO Label	Omicron
Pango lineage	BA.5
Next strain	22B
GISAID clade	GRA
Higher mutated strain in the spike protein	T19I, L24S, del25-27, del69-70, D142D, V213G, G339D, S371F, S373P, S375F, T376A, D405N, R408S, K417N, N440K, G446S, L452R, S477N, T478K, E484A, F486V, Q498R, N501Y, Y505H, T547K D614G, H655Y, N679K, P681H, N764K, D796Y, Q954H, N969K
Earliest detected	South Africa, Jan-2022
Higher infectivity	BA.5 dominated in April (72%) and in May (92%) in South Africa ^#^_._ BA.5 dominated in August (88.8%) and in September (83.1%) in USA *.
Lower risk of hospitalization, ICU admission, and mortality	Adjusted hazard ratio [aHR] 1.12; 95% confidence interval [CI]: 0.93; 1.34 [49].
Immune escape	BA.5 could further escape from the immunity induced by vaccination and BA.1 or BA.2 prior infections.

# Available online: https://www.nicd.ac.za/latest-confirmed-cases-of-covid-19-in-south-africa/ (accessed on 28 July 2022). * Available online: https://covid.cdc.gov/covid-data-tracker/#variant-proportions (accessed on 14 August 2022).

## Data Availability

The data supporting this study’s findings are available from the corresponding author upon reasonable request.
